# Evaluation of a commercially‐available block for spatially fractionated radiation therapy

**DOI:** 10.1120/jacmp.v11i3.3163

**Published:** 2010-04-26

**Authors:** Courtney Buckey, Sotirios Stathakis, Ken Cashon, Alonso Gutierrez, Carlos Esquivel, Chengyu Shi, Nikos Papanikolaou

**Affiliations:** ^1^ Departments of Radiology and Radiation Oncology, Cancer Therapy and Research Center University of Texas Health Science Center at San Antonio San Antonio TX 78229 USA; ^2^ Dot Decimal Inc. Sanford Florida 32771 USA

**Keywords:** spatially fractionated radiotherapy, GRID therapy, compensators

## Abstract

In this paper, we present the dosimetric characteristics of a commercially‐produced universal GRID block for spatially fractioned radiation therapy. The dosimetric properties of the GRID block were evaluated. Ionization chamber and film measurements using both Kodak EDR2 and Gafchromic EBT film were performed in a solid water phantom to determine the relative output of the GRID block as well as its spatial dosimetric characteristics. The surface dose under the block and at the openings was measured using ultra thin TLDs. After introducing the GRID block into the treatment planning system, a treatment plan was created using the GRID block and also by creating a GRID pattern using the multi‐leaf collimator. The percent depth doses measured with film showed that there is a shift of the $d_{\max}$ towards shallower depths for both energies (6 MV and 18 MV) under investigation. It was observed that the skin dose at the GRID openings was higher than the corresponding open field by a factor as high as 50% for both photon energies. The profiles showed the transmission under the block was in the order of 15–20% for 6 MV and 30% for 18 MV. The MUs calculated for a real patient using the block were about 80% less than the corresponding MUs for the same plan using the multileaf collimator to define the GRID. Based on this investigation, this brass GRID compensator is a viable alternative to other solid compensators or MLC‐based fields currently in use. Its ease of creation and use give it decided advantages. Its ability to be created once and used for multiple patients (by varying the collimation of the linear accelerator jaws) makes it attractive from a cost perspective. We believe this compensator can be put to clinical use, and will allow more centers to offer GRID therapy to their patients.

PACS number: 87.53.Mr

## I. INTRODUCTION

Spatially fractionated radiation therapy via GRID therapy is utilized to treat large tumors by irradiating the volume through isolated small openings.^(^ ^1^ ^–^ ^3^ ^)^ The technique has shown high efficacy for bulky tumors receiving doses of 10 to 20 Gy in a single fraction.^(^ ^2^ ^)^ The biological mechanisms of this technique are not fully understood, although several theories have been proposed.^(^ ^2^ ^)^ For patients who undergo GRID therapy followed by conventional fractionated radiation therapy, it has been speculated that the high cell kill of the GRID fraction induces re‐oxygenation, which improves the outcome of the subsequent conventional radiation therapy.^(^ ^4^ ^)^ Furthermore, despite the large single fraction, minimal skin radiation side effects are observed with spatially fractionated radiotherapy since only a small portion of the skin is exposed.

Historically, dense GRID collimators used for the radiation delivery were attached to the gantry.^(^ ^1^ ^–^ ^6^ ^)^ Although the results have been promising, the number of institutions practicing GRID therapy are very few. Many of the reasons for this hesitance have to do with the construction of the GRID itself. Difficulty lies in creating a GRID block that follows the beam divergence; that can be easily mounted and dismounted from the collimator; and the lack of understanding of the biological mechanisms behind GRID therapy. These difficulties make radiation oncologists skeptical about the use of spatially fractionated radiation therapy. Ha et al.^(^ ^7^ ^)^ showed that multileaf collimators (MLCs) can be used to deliver spatially fractionated radiotherapy with dosimetric properties at least as good as the ones obtained by using cerrobend block GRIDs.

In this study, we exploit the use of a commercially‐produced prototype brass GRID. This GRID has been manufactured such that it can be placed in the wedge tray slot, instead of the block tray. The GRID is made of the same material used for solid compensator IMRT. The use of such a compensator can solve the problem of manufacturing, since it can have wide commercial availability, has very high accuracy, weighs less than other GRID blocks currently in use, and can be machined to be universally applicable or patient‐specific. Before we can use this GRID block clinically, its dosimetric properties need to be determined. In this work, we study the dosimetric properties of the brass GRID for two high‐energy photon beams, 6 and 18 MV. The skin dose will also be measured for each energy.

## II. MATERIALS AND METHODS

**Figure 1:**
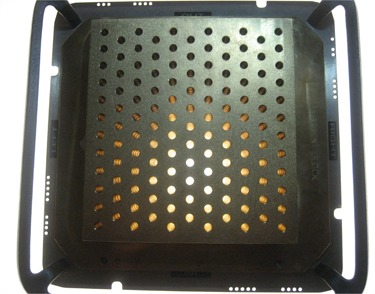
Photo showing the GRID block.

A GRID block was created by milling a block of brass. The GRID block was manufactured by .decimal (.decimal Inc., Sanford, FL). It was made so that it could irradiate a maximum field size of $25\times 25\;{\text{cm}}^{2}$. It is secured on a metal tray, and can be placed at the same location as a regular IMRT solid compensator. The height of the block is 7.62 cm and it weighs 15.8 kg. The holes are arranged so they form a hexagonal array (similar to a honeycomb) and the holes' centers are 2.0 cm apart. The holes are divergent, and each hole projects a 1.0 cm circular field at the isocenter (Fig. 1).

Measurements for the characterization of the dosimetric properties of the GRID were performed using a Varian Clinac 23Ex linear accelerator (Varian Medical Systems, Palo Alto, CA). The measurements were performed for both energies available with this linac, namely 6 MV and 18 MV. All measurements were performed in a solid water phantom (CNMC Inc, Nashville, TN) placed so that the source to surface distance (SSD) was 100.0 cm. Kodak EDR2 films were placed in the phantom at the depths of $d_{\max}$ 5.0 cm and 10.0 cm, perpendicular to the beam axis. Another set of film measurements were performed with the films parallel to the beam axis. The first set of film measurements were used to extract information about the lateral profiles, while the latter were used to obtain percent depth dose (PDD) curves. The VIDAR scanner Pro16 (Vidar systems Corp., Herndon, VA) was used for digitizing the films, and the RIT113 software (Radiological Imaging Technology, Colorado Springs, CO) was used for film analysis.

**Figure 2:**
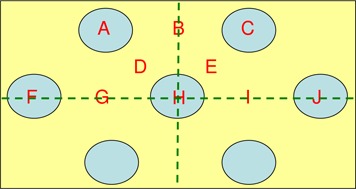
Locations of the surface dose measurements with respect to the GRID design. Hole centers are 2 cm apart.

Ultrathin TLDs were used to evaluate the skin dose, under the holes and the blocked areas. Calibration factors were obtained for each energy, and measurements were repeated to increase accuracy. Figure 2 shows the measurement positions, with respect to the central hole(point H).

Ion chamber measurements were obtained to determine the output factors of the GRID. The output factors were determined for both photon energies using a pinpoint PTW (PTW, Germany) ionization chamber of 0.016 cc effective volume inside a solid water phantom. Measurements at $d_{\max}$ under the central GRID hole were collected for the determination of the output factors while changing the field size defined by the X and Y jaws. Finally, the measurements were normalized against the reading at the same depth of a $10\times 10\;{\text{cm}}^{2}$ field.

The GRID design was imported into the ${\text{Pinnacle}}^{3}$ treatment planning system (TPS) in order to be used clinically. This included the creation of a brass compensator that can be placed in the beam. In our department, only the 6 MV photon beam is currently commissioned for solid compensator treatments and, hence, we are limited to using only the lower energy for clinical treatments. A case study was created using the brass GRID and an analogous MLC‐based GRID, for a patient that presented to our clinic with stage IVC head and neck squamous cell carcinoma. The patient was to be treated with a single fraction of spatially fractionated radiotherapy of 15.0 Gy prescribed to the isocenter, which is approximately centered in the tumor volume, and is underneath the central axis hole in the compensator block. This was then to be followed by 5 fractions of conformal, unmodulated beams, each fraction delivering 6.0 Gy. The initial plan using the GRID block was composed of a single beam of the 6 MV photon energy. The delivery of the treatment was validated prior to the treatment using point and film measurements. Finally, an analogous plan was created using the multileaf collimator (MLC) for the delivery of the spatially fractioned treatment. The two methods of delivery of spatially fractionated treatments were compared in terms of efficacy, number of MUs, and isodose distributions.

## III. RESULTS & DISCUSSION

**Figure 3:**
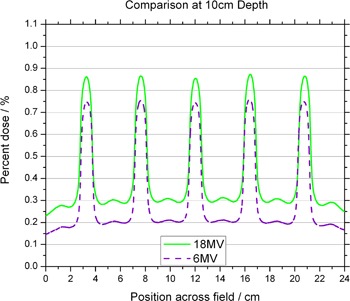
Comparison of the off‐axis profiles between the 6 MV and 18 MV photon beams at 10 cm depth. Profiles are normalized to the central axis dose measured at a depth of $d_{\max}$ for their respective energy.

A 3D representation of the isodose distribution of the spatially fractionated beam was generated. The distribution was reconstructed from film measurements inside a water equivalent phantom. The film was placed in the phantom at a depth of 10.0 cm, perpendicular to the beam axis. The GRID was inserted in the beam's path and the jaws were set to $25\times 25\;{\text{cm}}^{2}$, which is the maximum allowable field size using this particular GRID compensator. The measurements using film revealed profiles that have an obvious peak and valley pattern, with transmissions through the solid portion of the block of approximately 15%–20% for 6 MV and 30% for 18 MV (Fig. 3).

**Figure 4:**
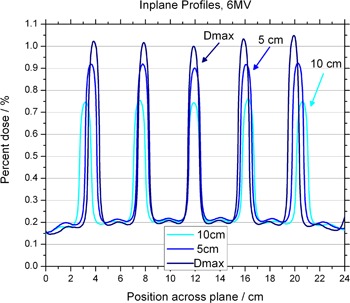
Off‐axis profiles at depths of $d_{\max}$, 5 cm and 10 cm, for the 6 MV photon beam. Profiles are normalized to the central axis dose measured at a depth of $d_{\max}$.

**Figure 5:**
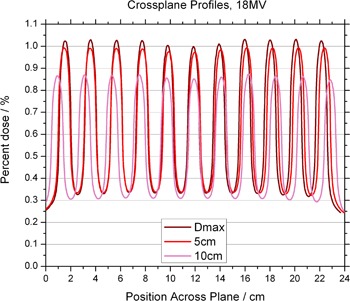
Off‐axis profiles at depths of $d_{\max}$, 5 cm and 10 cm, for the 18 MV photon beam. Profiles are normalized to the central axis dose measured at a depth of $d_{\max}$.

**Figure 6:**
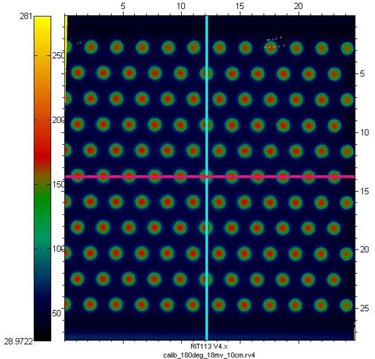
Film taken at 10 cm in a solid water phantom, with the pink (horizontal) line representing the cross‐plane profiles and the blue (vertical) line representing the in‐plane profiles.

Figures 4 and 5 show in‐plane and cross‐plane profiles for films taken at depths equal to $d_{\max}$, 5 cm and 10 cm. A recently acquired calibration curve was applied to all films before analysis. Figures 3 to 5 have been normalized to the central axis dose measured at a depth of $d_{\max}$. The ratio of peak to valley doses is seen to be a function of depth for both 6 MV and 18 MV. The maximum values appear for measurements taken at $d_{\max}$. The in‐plane profiles have fewer peaks and valleys than the cross‐plane profiles because of the pattern of the holes. The perturbations seen between peaks of the in‐plane profile are due to the honeycomb pattern of the holes — a peak appears in the area “between” two neighboring holes. This pattern can be seen in Fig. 6, which shows a film taken at 10 cm in a solid water phantom, with the pink (horizontal) line representing the cross‐plane profiles and the blue (vertical) line representing the in‐plane profiles.

**Figure 7:**
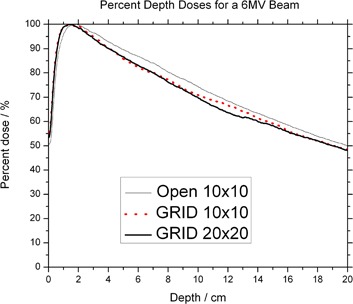
Percent depth dose of the 6 MV photon beam.

**Figure 8:**
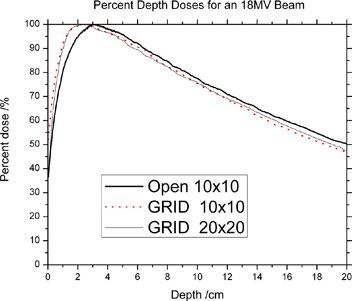
Percent depth dose for the 18 MV photon beam.

The PDDs are less penetrating than their open‐field counterparts, for both 6 MV and 18 MV (Figs. 7 and 8). It is also observed that the depth of maximum dose is shifted to shallower depth for both energies. These results agree with published data showing that $d_{\max}$ shifts toward the surface as field sizes are made smaller. Effectively, the surface dose is higher along the GRID opening when comparing to the open fields. The increase is on the order of 50% when compared to a $10\times 10\;{\text{cm}}^{2}$ field. The surface dose under the blocked parts is lower than the open fields (Table 1).

**Table 1:** Surface dose measurements normalized to the dose at $d_{\max}$ of $10\times 10\;{\text{cm}}^{2}$. Values are percents.

	*6x*	*18x*
Surface of $10\;\times \;10\;{\text{cm}}^{2}$	18.3±0.9	12.5±0.6
Average Hole Dose at Surface (A,C,F,H,J)	27.1±1.7	19.9±1.2
Average (Near) Blocked Dose at Surface (D,E)	6.7±0.3	12.9±1.3
Average (Far) Blocked Dose at Surface (G,I)	7.1±1.0	12.8±1.1

With MLC GRIDs, various openings and open‐to‐block areas can be designed with minimum effort and in very little time. One advantage of utilizing MLCs over solid blocks is the ease of designing multiple GRID patterns. A significant drawback with the MLC approach is the increased number of monitor units required, as compared to the block type GRIDs which have been previously used. The increased number of monitor units has a direct impact on the surface dose and leakage through the MLCs. Table 2 shows the results in a real‐life situation, that of our head and neck patient. The prescription dose was 15 Gy to the isocenter, and the MUs for the MLC delivery were higher by 529%.

**Table 2:** Comparison of treatment time (MU) between MLC and compensator treatments

*Prescription*	*15 Gy*
MLC (MU)	17028 (18 fields of 946)
Compensator Grid (MU)	2874 (3 fields of 958)
Difference	529%

**Figure 9:**
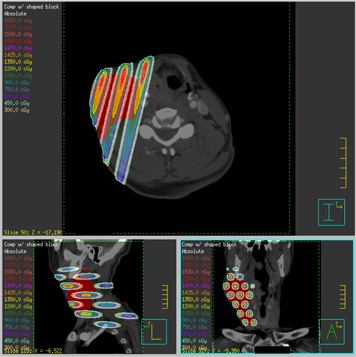
Transverse, coronal and sagittal views of the isodose distribution of the GRID compensator block plan.

**Figure 10:**
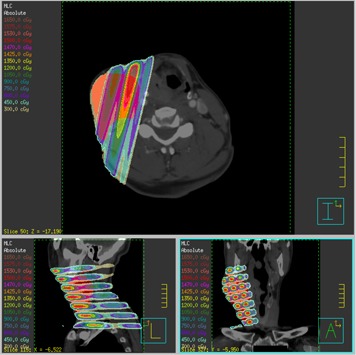
Transverse, coronal and sagittal views of the isodose distribution of the GRID MLC plan.

**Figure 11:**
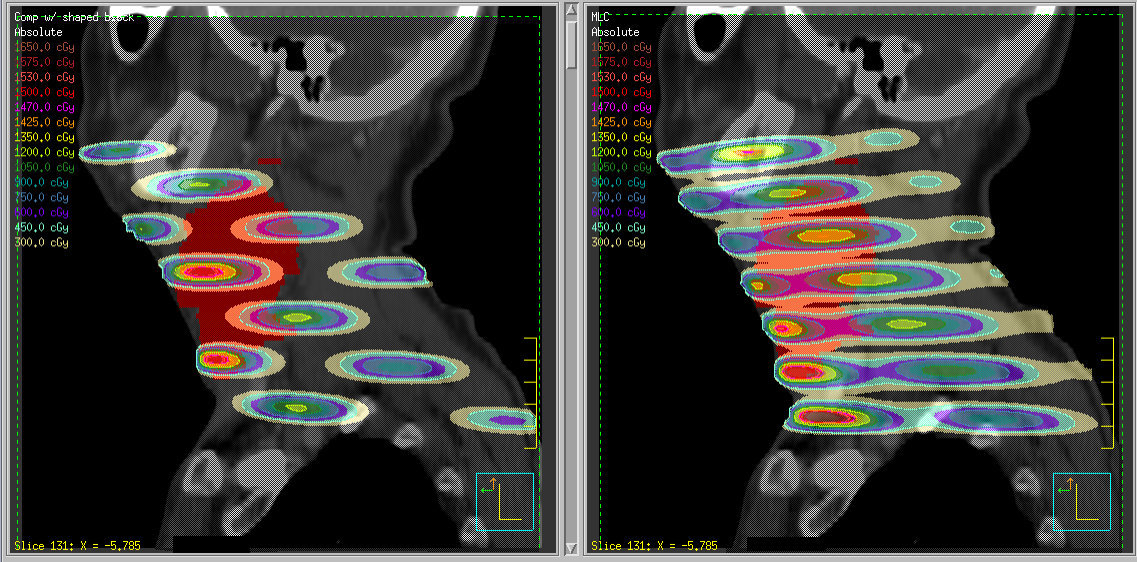
Sagittal comparison of the compensator block (left) and MLC‐based field (right).

**Figure 12:**
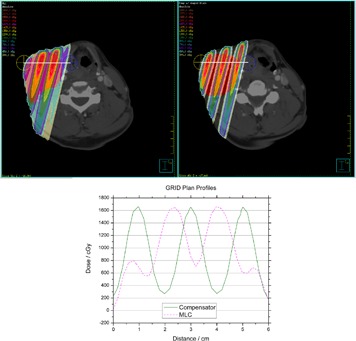
Profiles through a high‐dose region of the MLC‐based plan (left) and the compensator‐based plan (right). Profiles are taken along the white line from the yellow marked point to the blue marked point.

The isodose distributions for the compensator GRID can be seen in Figs. 9–12. The 3‐view window from Pinnacle is shown for the GRID compensator block in Fig. 9, and the MLC‐based plan in Fig. 10. A more detailed image of just the sagittal view is shown in Fig. 11. From these three figures, one can see that the dose from the compensator block is well collimated, with low doses under the blocked areas. In comparison, the MLC delivery reflects the leakage inherent in MLC‐based fields, which smears out the low dose regions — potentially negating the spatial fractionation that we intend to exploit. The sagittal view also shows that for the MLC‐based plan, the prescription dose is sometimes delivered to areas outside the PTV, whereas the compensator‐based plan seems to better localize the high doses.

Figure 12 shows profiles through a high‐dose region of the MLC‐based plan and compensator block plan. The profiles are taken along a line from the yellow marked point to the blue marked point. They were selected to give the best representation of each method's peak‐to‐valley ratio, and they are not at the same position in the patient. The maximum values for each field are nearly identical: 1650 cGy. The MLC‐based field's valley dose is 700 cGy, which results in a ratio of 2.36, while the compensator block's valley dose is 250 cGy, resulting in a ratio of 6.6. Other investigators have noted that when delivering a single dose of 15–20 Gy to the open regions, it is desirable for the dose under the blocked regions to be kept below what would be used in a conventional fractionation scheme.^(^ ^7^ ^)^ Based on that criterion, the GRID block would be the better choice as it gives a lower dose. It also seems reasonable to believe that method with the higher the peak‐to‐valley ratio (the greater degree of spatial fractionation) would be the preferred delivery method.

If photon beams higher than 10 MV are used, neutron contamination must be taken into consideration. The monitor units required for a typical prescription of 15 Gy to $d_{\max}$ along the central axis, using MLC delivery, are on the order of 15,000 to 20,000 MU.^(^ ^8^ ^)^ The large number of MUs are attributed to the fact that, although the output along the central axis is on the order of 0.6 to 0.9 of a $10\times 10\;{\text{cm}}^{2}$ open field, the MUs are dictated by the number of segments required to cover the target. From the literature, MLC GRID treatments were reported to be 5 times longer than those with a Cerrobend GRID — a value in agreement with the time measured in our experiments.^(^ ^7^ ^)^

## IV. CONCLUSIONS

Based on the present investigation, this brass GRID compensator is a viable alternative to the presently‐used compensators or MLC‐based fields currently in use. Its ease of creation and use give it decided advantages. It needs to be created and purchased only once, but can be used to treat a virtually unlimited amount of patients simply by varying the collimation of the linear accelerator jaws. This makes it attractive from a cost perspective. The reduction in monitor units delivered as compared to an MLC‐based field decreases the amount of time the patient must be immobilized for treatment. We believe this compensator can be put to clinical use, and will allow more centers to offer GRID therapy to their patients.

## ACKNOWLEDGEMENTS

.decimal Inc. supported this investigation.
